# Kefir peptides attenuate atherosclerotic vascular calcification and osteoporosis in atherogenic diet-fed *ApoE* ^−/−^ knockout mice

**DOI:** 10.3389/fcell.2023.1158812

**Published:** 2023-04-06

**Authors:** Gary Ro-Lin Chang, Wei-Yuan Cheng, Hueng-Chuen Fan, Hsiao-Ling Chen, Ying-Wei Lan, Ming-Shan Chen, Chih-Ching Yen, Chuan-Mu Chen

**Affiliations:** ^1^ Department of Pediatrics, Department of Medical Research, Tungs’ Taichung Metroharbor Hospital, Taichung, Taiwan; ^2^ Department of Life Sciences, and Ph.D. Program in Translational Medicine, National Chung Hsing University, Taichung, Taiwan; ^3^ Department of Rehabilitation, Jen-Teh Junior College of Medicine, Miaoli, Taiwan; ^4^ Department of Biomedical Sciences, and Department of Bioresources, Da-Yeh University, Changhwa, Taiwan; ^5^ Division of Pulmonary Biology, Cincinnati Children’s Hospital Medical Center, University of Cincinnati, Cincinnati, OH, United States; ^6^ Department of Anesthesiology, Ditmanson Medical Foundation Chia-Yi Christian Hospital, Chia-Yi, Taiwan; ^7^ Department of Internal Medicine, China Medical University Hospital, and College of Healthcare, China Medical University, Taichung, Taiwan; ^8^ The iEGG and Animal Biotechnology Center, National Chung Hsing University, Taichung, Taiwan

**Keywords:** vascular calcification (VC), osteoporosis, kefir peptides (KPs), apolipoprotein E (APOE), atherosclerosis, hyperlipidemia, ox-LDL, inflammation

## Abstract

**Aims:** Vascular calcification (VC) and osteoporosis were previously considered two distinct diseases. However, current understanding indicates that they share common pathogenetic mechanisms. The available medicines for treating VC and osteoporosis are limited. We previously demonstrated that kefir peptides (KPs) alleviated atherosclerosis in high-fat diet (HFD)-induced apolipoprotein E knockout (*ApoE* ^*−/−*^) mice. The present study further addressed the preventive effects of KPs on VC and osteoporosis in *ApoE* ^*−/−*^ mice fed a high-cholesterol atherogenic diet (AD).

**Main methods:** Seven-week-old *ApoE* ^*−/−*^ and wild-type C57BL/6 mice were randomly divided into five groups (*n* = 6). The development of VC and osteoporosis was evaluated after AD feeding for 13 weeks in KP-treated *ApoE* ^*−/−*^ mice and compared to C57BL/6 and *ApoE* ^*−/−*^ mice fed a standard chow diet (CD).

**Key findings:** The results indicated that KP-treated *ApoE* ^*−/−*^ mice exhibited lower serum total cholesterol, oxidized low-density lipoprotein (ox-LDL), malondialdehyde (MDA) levels, and serum alanine aminotransferase (ALT), aspartate aminotransferase (AST) and creatine kinase (CK) activities, which suggested that KPs prevented hyperlipidemia and possible damages to the liver and muscle in *ApoE* ^*−/−*^ mice. KPs reduced serum tumor necrosis factor-α (TNF-α) and the local expression of TNF-α, IL-1β, and macrophage-specific CD68 markers in aortic tissues, which suggested that KPs inhibited inflammatory responses in AD-fed *ApoE* ^*−/−*^ mice. KPs reduced the deposition of lipid, collagen, and calcium minerals in the aortic roots of AD-fed *ApoE* ^*−/−*^ mice, which suggested that KPs inhibited the calcific progression of atherosclerotic plaques. KPs exerted osteoprotective effects in AD-fed *ApoE* ^*−/−*^ mice, which was evidenced by lower levels of the bone resorption marker CTX-1 and higher levels of the bone formation marker P1NP. KPs improved cortical bone mineral density and bone volume and reduced trabecular bone loss in femurs.

**Significance:** The present data suggested that KPs attenuated VC and osteoporosis by reducing oxidative stress and inflammatory responses in AD-fed *ApoE* ^*−/−*^ mice. Our findings contribute to the application of KPs as preventive medicines for the treatment of hyperlipidemia-induced vascular and bone degeneration.

## Introduction

Cardiovascular disease and osteoporosis are two important issues in the aging population, and these conditions are more critical in patients with chronic renal failure, type 2 diabetes, dyslipidemia and inflammatory rheumatic diseases (Quaglino et al., 2020). Both diseases severely impact on daily physiological functioning, quality of life, and life expectancy. Atherosclerosis is the major cause of cardiovascular diseases, and its adverse consequences include further progression to vascular calcification (VC), stenosis, and total occlusion. Osteoporosis is the 4th most prevalent health issue worldwide, and it is characterized by low bone mass and degenerated bone structure, which increase the risk of fracture and reduce health status due to inactivity (Zeng et al., 2019). VC and osteoporosis were considered separately related to aging, menopause, diabetes mellitus, smoking, and a sedentary lifestyle, but recent findings showed that these conditions often occur simultaneously. VC is caused by the deposition of calcium minerals in primary blood vessels, and it is an actively regulated process that shares common mechanisms with bone formation (Durham et al., 2018; Quaglino et al., 2020). The contradictory connection between bone loss and VC is commonly referred to as the “calcification paradox” or the “bone-vascular axis” (Hjortnaes et al., 2010; Vassalle and Mazzone, 2016; Evenepoel et al., 2019). The underlying pathophysiology of this correlation is complicated and worthy of investigation.

Epidemiological and preclinical studies exhibit that hyperlipidemia is a major risk factor for atherosclerosis and associated cardiovascular diseases. Clinically, hyperlipidemia refers to higher plasma levels of cholesterol, triglycerides, and low-density lipoprotein (LDL)-cholesterol or a lower high-density lipoprotein (HDL)-cholesterol level than normal standards. High LDL-cholesterol-induced hyperlipidemia contributes to more than one-third of deaths due to ischemic heart diseases and ischemic stroke (Pirillo et al., 2021). The WHO estimated that the global prevalence of hypercholesterolemia in adults >25 years was approximately 39% in 2018, but this ratio has continued to rise in recent years (World Health Organization, 2022). A growing number of reports revealed a significant correlation of hyperlipidemia with osteoporosis (Wong et al., 2016; Papachristou et al., 2018), which can be partly explained by increased oxidized cholesterol (ox-LDL) in the blood. Ox-LDL is considered as a potent inducer of the oxidative stress and inflammation that cause atherosclerosis, and this dynamic process further progresses to form atheroma and calcification inside the arterial wall (Mitra et al., 2011). Concurrently, ox-LDL had been reported to enhance the production of receptor activator of nuclear factor-kappa B ligand (RANKL) in human T lymphocytes (Graham et al., 2009) and osteoblast-like cells (Maziere et al., 2013), and was shown to inhibit phosphate (Pi) signaling and Pi-induced mineralization in rat osteoblasts by generating oxidative stress (Maziere et al., 2010). These studies support the idea that vascular calcification promotes bone loss. On the contrary, osteoblasts are known to release abundant regulatory factors (e.g., RANKL (Panizo et al., 2009), osteopontin (Cho et al., 2009), and osteoprotegerin (OPG) (Rochette et al., 2019)) that also act on blood vessels, and the common agents (estrogen (Wu et al., 2020), parathyroid hormone (Shao et al., 2003), *etc.*) for osteoporosis also accompany an inhibition of VC. Although paradoxically, VC and osteoporosis are currently considered to have a common etiology. Most recently, the fibroblast growth factor-23 (FGF23)/Klotho axis has been demonstrated to play the underlying connection between VC and osteoporosis (Wei et al., 2021).

Clinically, statins are the most common drugs used to control cholesterol levels and cardiovascular diseases. However, 30%–40% of patients remain at risk of atherosclerosis progression. Intensive statin treatment is associated with new-onset diabetes mellitus, hepatotoxicity, myopathy and cognitive decline (Ward et al., 2019; Kim et al., 2020). Most guidelines for osteoporosis recommend adequate calcium intake for prevention and treatment, but too much supplemental calcium may accelerate VC and increase cardiovascular events, especially in patients with chronic renal failure (Bolland et al., 2010; Hill Gallant et al., 2016). Considering the unmet need of the present medicines for the treatment of VC and osteoporosis simultaneously, a novel therapeutic strategy based on a holistic understanding of the bone-vascular axis is warranted to further solve the threat of cardiovascular diseases and osteoporosis.

Kefir originated in the Caucasian mountains, and it is an acidic and alcoholic beverage produced by milk fermentation with kefir grains. The microbial composition of kefir grains includes a variety of probiotics, such as lactobacteria, acetic acid bacteria, and yeasts, which degrade milk proteins into abundant bioactive peptides called kefir peptides (KPs) that exert multiple functions, such as antioxidation, anti-inflammation, and immunomodulation (Rosa et al., 2017). We previously demonstrated the bone-protective effects of KPs in a variety of osteoporotic animal models and osteoporotic patients (Chen et al., 2015; Tu et al., 2015; Tu et al., 2020; Chang et al., 2022; Yen et al., 2022) and reported the anti-atherosclerotic activity of KPs in *ApoE* ^*−/−*^ mice fed high-fat diets (Tung et al., 2020). The present study further elucidated the preventive effects of KPs on VC and osteoporosis in *ApoE* ^*−/−*^ mice. The present data support the potential of KPs as a preventive medicine for the clinical management of VC and osteoporosis. To the best of our knowledge, this report is the first study to show the benefits of KPs against simultaneous VC and osteoporosis.

## Materials and methods

### Kefir peptides

The KP powder (KEFPEP^®^) used in the present study was manufactured by Phermpep Biotech. Co., Ltd. (Taichung, Taiwan) as described previously (Chen et al., 2015). The peptide contents in 100 g of KP powder was 23.1 g as determined using the o-phthalaldehyde method. The calories in 100 g of KP powder were 487 kcal (Tu et al., 2015; Tung et al., 2018). The KP solutions for oral administration were freshly prepared in phosphate-buffered saline (PBS).

### Mouse model

To avoid the disturbance of estrogen and the estrous cycle in the animal model establishment, only male mice were used. In this study, male C57BL/6 (B6) and *ApoE* ^*−/−*^ mice (B6.129P2-Apoetm1Unc/J) were purchased from National Applied Research Laboratories (Taipei, Taiwan) and the Jackson Laboratory (Bar Harbor, ME, United States), respectively. All of the *ApoE* ^*−/−*^ mice used have been characterized as homologous genotypes. All mice were housed in an air-conditioned animal room with a 12-h cycle of light and darkness and proper indoor temperature (20°C–23°C) and humidity (50%–60%). Mice had access to water and a standard chow diet (CD, 3.23 kcal/g) (Atromin^®^-1324, Lage, Germany) or a high-cholesterol (1.25%) atherogenic diet (AD; 4.05 kcal/g) (TestDiet^®^-57BB, St. Louis, MO, United States) *ad libitum*. The Institutional Animal Care and Use Committee of National Chung Hsing University, Taichung, Taiwan approved the animal experiments (No. 104-076^R^) which were performed by fully educated and trained laboratory members to ensure animal welfare and reduce unnecessary animal suffering.

Seven-week-old mice were randomly divided into five groups (*n* = 6) as follows: (1) B6 mice fed CD (WT + CD); (2) *ApoE* ^*−/−*^ *mice* fed CD (mock + CD); (3) *ApoE* ^*−/−*^ mice fed AD (mock + AD); (4) *ApoE* ^*−/−*^ mice fed AD and orally treated with 328 mg/kg low-dose KPs (KPs-L + AD); and (5) *ApoE* ^*−/−*^ mice fed AD and orally treated with 656 mg/kg high-dose KPs (KPs-H + AD). The low dose used for mice corresponded to the same amount of KPs used in our previous clinical cohort study for osteoporotic patients (*n* = 40) (Tu et al., 2015), whereas the high dosage was determined by the 2-fold quantity of KPs used for humans. The *ApoE* ^*−/−*^ mice (groups 3–5) were switched to AD at 6 weeks of age, followed by daily oral gavage of 100 μL kPs or PBS at 7 weeks of age for a total of 13 weeks. Body weight and diet intake were recorded weekly. At the end of treatment, blood samples were collected *via* retro-orbital bleeding under isoflurane anesthesia for serological examination. Aortas and femurs were dissected and fixed in 10% neutral formalin for histological examination and micro-CT scanning, respectively.

### Serological examination

Total cholesterol, triglycerides, and the enzymatic activities of alanine aminotransferase (ALT), aspartate aminotransferase (AST), and creatine kinase (CK) were measured using IDEXX dry-slide technology and the VetTest^®^ chemistry analyzer (IDEXX Laboratories Inc., Westbrook, ME, United States). The malondialdehyde (MDA) level was measured using a TBARS assay kit (No.10009055; Cayman Chemical, Ann Arbor, MI, United States). The serum level of tumor necrosis factor-alpha (TNF-α) was determined using a mouse-specific ELISA kit (ab208348; Abcam, Cambridge, United Kingdom). The serum levels of oxidized low-density lipoprotein (ox-LDL) (SEA527Mu), procollagen I N-terminal propeptide (P1NP) (SEA957Mu), and cross linked C-telopeptide of type I collagen (CTX-1) (CEA665Mu) were determined using mouse-specific ELISA kits from Cloud-Clone Corp (Katy, TX, United States).

### Histological examination

After overnight fixation in 10% neutral formalin, the aortic samples were embedded in OCT frozen section compound (Leica REF 3801480, Wetzlar, Hesse, Germany), and cut into 10–12 µm sections for hematoxylin and eosin (H&E), Oil-red O, and Masson’s trichrome staining as described previously (Tung et al., 2020). H&E staining was performed to examine the sizes of atherosclerotic plaques, Oil-red O staining was performed to assess lipid deposition, and Masson’s trichrome staining was performed to identify the collagen fiber contents in the tunica intima of the aortic root. Quantification was performed by inspecting 4 (slides 3–6) of 9 serial sections of the aortic root tissues from each mouse, as described previously (Tung et al., 2020). The deposition of calcium minerals in atherosclerotic lesions of the aortic roots was identified using a von Kossa stain kit (ab150687; Abcam).

### Immunohistochemistry (IHC)

IHC was performed using a Novolink Polymer Detection Systems kit (RE7280-K) from Leica Biosystems (Buffalo Grove, IL, USA), and a primary antibody against mouse CD68 was purchased from Abcam (ab955, 1:100 dilution). Pretreatment and subsequent blocking, antibody incubation, washing, and the development of peroxidase activity were performed according to the manufacturer’s instructions. This method was used to identify the expression of the pan-macrophage marker CD68 in atherosclerotic lesions of the aortic roots.

### Western blotting

Frozen samples of aortas were homogenized in 1x RIPA buffer (50 mM Tris-HCl, 150 mM NaCl, 1% NP-40, 0.25% sodium deoxycholate, 5 mM EDTA, 0.1% SDS) with proteinase inhibitors and quantitated using the BCA method. The protein lysates (50 μg) were loaded, separated in a 10% SDS-polyacrylamide gel, and transferred onto a PVDF membrane. The membrane was blocked at room temperature for 2 h with 5% BSA then incubated overnight at 4°C with appropriate dilutions of α-tubulin (Cell Signaling #2125S, 1:3000), IL-1β (Cell Signaling #12242S, 1:5000), and TNF-α (Proteintech #60291-1-1g, 1:2000) antibodies. The membrane was sequentially washed three times with TBST (10 mM Tris-HCl, 150 mM NaCl, 0.05% Tween 20), incubated with diluted HRP-conjugated secondary antibody (1:10,000), and washed three times with TBST. The protein signals were developed by adding highly sensitive HRP substrate and detected using the ImageQuant LAS 4000 mini luminescent image analyzer (GE Healthcare, Chicago, IL, United States).

### Microcomputed tomography (μ-CT)

Mouse femurs were scanned at 9-μm resolution using a SkyScan 1076 μ-CT scanner (Bruker, Belgium). The resultant 2-dimensional (2D) images were subjected to manual extraction of the volume of interest (VOI) of the bone regions to compute bone mineral density (BMD), the structural indices of trabecular (Tb) bones (volume fraction (Tb.BV/TV), number (Tb.N), thickness (Tb.Th), separation (Tb.Sp), pattern factor (Tb.Pf) and structure model index (SMI)), and the structural indices of cortical (Ct) bones (volume (Ct.BV), volume fraction (Ct.BV/TV), and bone surface (Ct.BS/BV)), as previously described (Chen et al., 2015; Lai et al., 2017; Tu et al., 2020). Three-dimensional (3D) images were reconstituted by superimposing 209 2-D images, which corresponded to a 1.8 mm thick area near the distal metaphysis.

### Statistical analysis

The present data were plotted in histograms (mean ± SD) or box plots (median, first/third quartiles, and minimum/maximum) using GraphPad Prism software. One-way ANOVA and Tukey’s *post hoc* test were used to analyze significant differences. Significant differences are indicated by the following symbols: *p < 0.05, **p < 0.01, and ***p < 0.001 compared to the WT + CD group; and ^#^ *p* < 0.05, ^##^ *p* < 0.01, and ^###^ *p* < 0.001 compared to the mock + AD group.

## Results

### Weekly changes in mouse body weight, diet, and energy intake

At week 0, CD-fed *ApoE^−/−^* mice (mock + CD) had a lower mean body weight than CD-fed B6 mice (WT + CD) (*p* < 0.001, Supplementary Figure S1A). The mean body weights of AD-fed *ApoE^−/−^* mice (mock + AD, KPs-L + AD, and KPs-H + AD) were significantly lower than the CD-fed B6 and *ApoE^−/−^* mice (*p* < 0.001, Supplementary Figure S1A). The trend in the changes in body weight was only slightly affected by the differences in diets and the oral treatment with KPs throughout the study, and the final weight gain in each group was not significantly affected (Supplementary Figure S1B). Diet intake did not intensely fluctuate during the experiment (Supplementary Figure S1C). The AD-fed *ApoE^−/−^* mice showed higher caloric intakes than the CD-fed B6 and *ApoE^−/−^* mice from week 7 (*p* < 0.001, Supplementary Figure S1D).

### KPs decrease serum cholesterol, ox-LDL, MDA, TNF-α, AST and CK levels in AD-fed *ApoE^−/−^* mice

**FIGURE 1:**
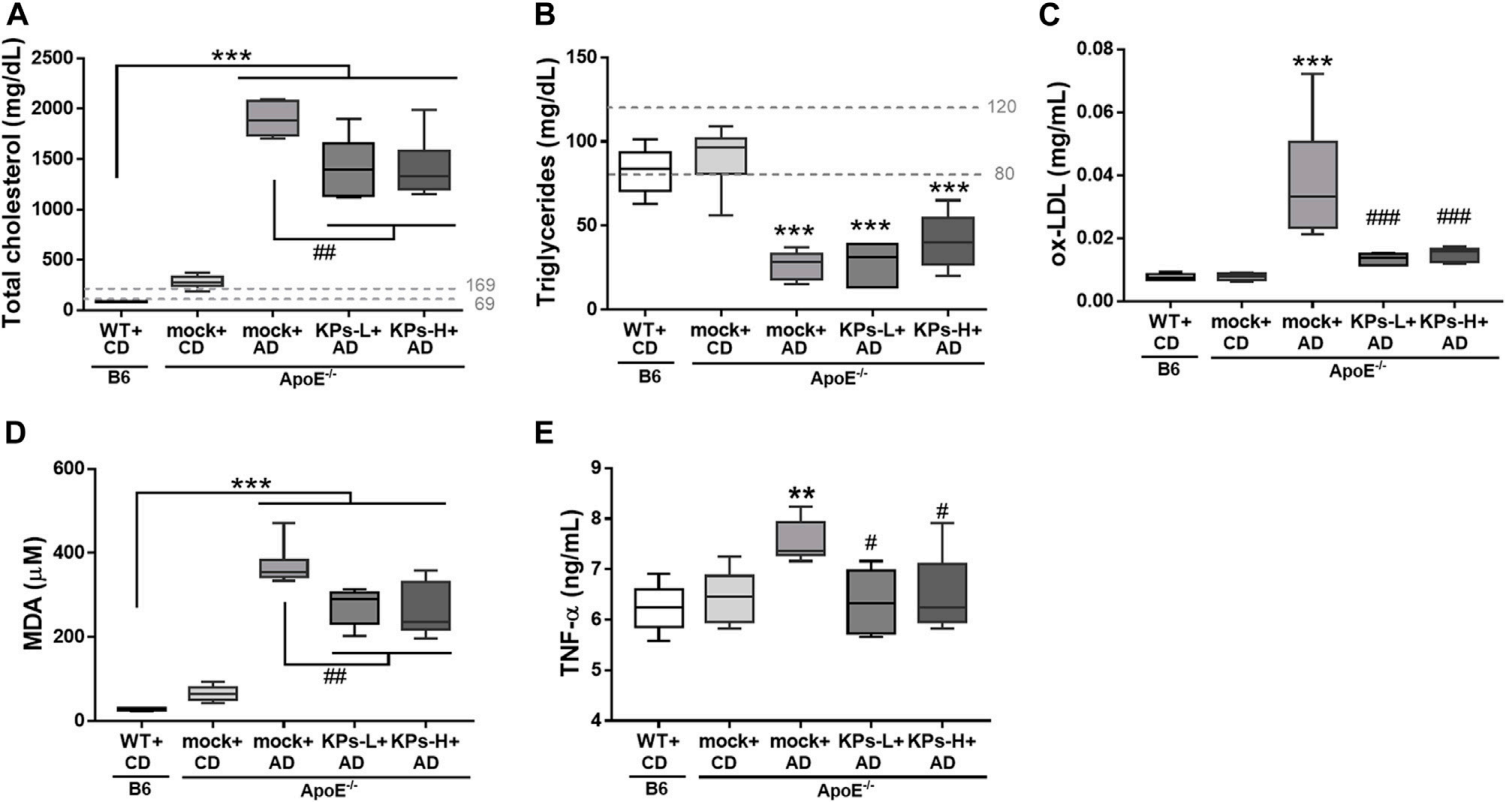
Effects of KPs on serum **(A)** cholesterol, **(B)** triglyceride, **(C)** ox-LDL, **(D)** MDA, **(E)** TNF-α. The dashed lines indicate the normal ranges of target serum markers in mice. Statistical signs (*n* = 6): *p < 0.05, **p < 0.01, ***p < 0.001 vs. WT + CD/B6, and ^#^ *p* < 0.05, ^##^ *p* < 0.01, ^###^ *p* < 0.001 vs. mock + AD/*ApoE* ^*−/−*^.

As shown in Figure 1A, the CD-fed *ApoE^−/−^* mice showed higher serum total cholesterol levels than the CD-fed B6 mice, but the difference was not significant, and both groups remained within the normal serum cholesterol range of mice. However, serum total cholesterol levels significantly increased beyond the normal range in the AD-fed *ApoE^−/−^* mice (*p* < 0.001). Oral administration of KPs decreased serum total cholesterol levels to a significant extent (*p* < 0.01), but these levels remained above the normal range. The AD-fed *ApoE^−/−^* mice showed lower serum triglyceride levels than the CD-fed B6 and *ApoE^−/−^* mice (*p* < 0.001, Figure 1B). Although the serum triglyceride level was slightly elevated in KPs-H + AD group, KP treatment did not alter the reduction in serum triglyceride levels.

Serum levels of potent proatherosclerotic mediator ox-LDL were measured (Figure 1C). In contrast to CD-fed B6 and *ApoE^−/−^* mice, AD induced a relatively higher serum ox-LDL level in *ApoE^−/−^* mice (*p* < 0.001). However, this increase was not observed in KP-treated *ApoE^−/−^* mice (*p* < 0.001). KP-treated *ApoE^−/−^* mice exhibited a comparable ox-LDL level to normal B6 mice.

MDA is the most common lipid marker of oxidative stress (Abedi et al., 2023). In this study, MDA levels were markedly high in the AD-fed *ApoE^−/−^* mice (*p* < 0.001, Figure 1D). However, oral KP administration significantly inhibited the increase in serum MDA, but the final MDA levels in KP-treated *ApoE^−/−^* mice were not reduced to the levels of normal B6 mice (Figure 1D).

AD-fed *ApoE^−/−^* mice showed increased low-grade systemic TNF-α compared to normal CD-fed B6 and *ApoE^−/−^* mice, but this change was not seen in KP-treated *ApoE^−/−^* mice (*p* < 0.05, Figure 1E).

Biomarkers for liver function, such as ALT (Supplementary Figure S2A) and AST (Supplementary Figure S2B), were examined. Serum levels of ALT and AST were indistinguishabe between CD-fed B6 and *ApoE^−/−^* mice, which had a normal AST/ALT ratio of approximately 1 (Supplementary Figure S2C). However, a notable increase in AST (beyond the normal range, *p* < 0.001) and a slight increase in ALT were detected in the AD-fed *ApoE^−/−^* mice, which led to a significant increase in their serum AST/ALT ratio (median = 2.42, *p* < 0.05). KPs prevented the increase in serum ALT and AST levels, particularly AST, and eventually caused a decrease in the AST/ALT ratio in the KPs-L + AD (median = 1.84) and KPs-H + AD (median = 2.11) groups.

AD induced a very high CK level in *ApoE^−/−^* mice compared to normal CD-fed B6 and *ApoE^−/−^* mice (*p* < 0.001, Supplementary Figure S2D). This biomarker for muscle damage was also substantially inhibited by low and high doses of KP compared to *ApoE^−/−^* mice without KP treatment (*p* < 0.001, Supplementary Figure S2D).

### KPs attenuate atherosclerotic vascular calcification of the aortic root in AD-fed *ApoE^−/−^* mice

**FIGURE 2:**
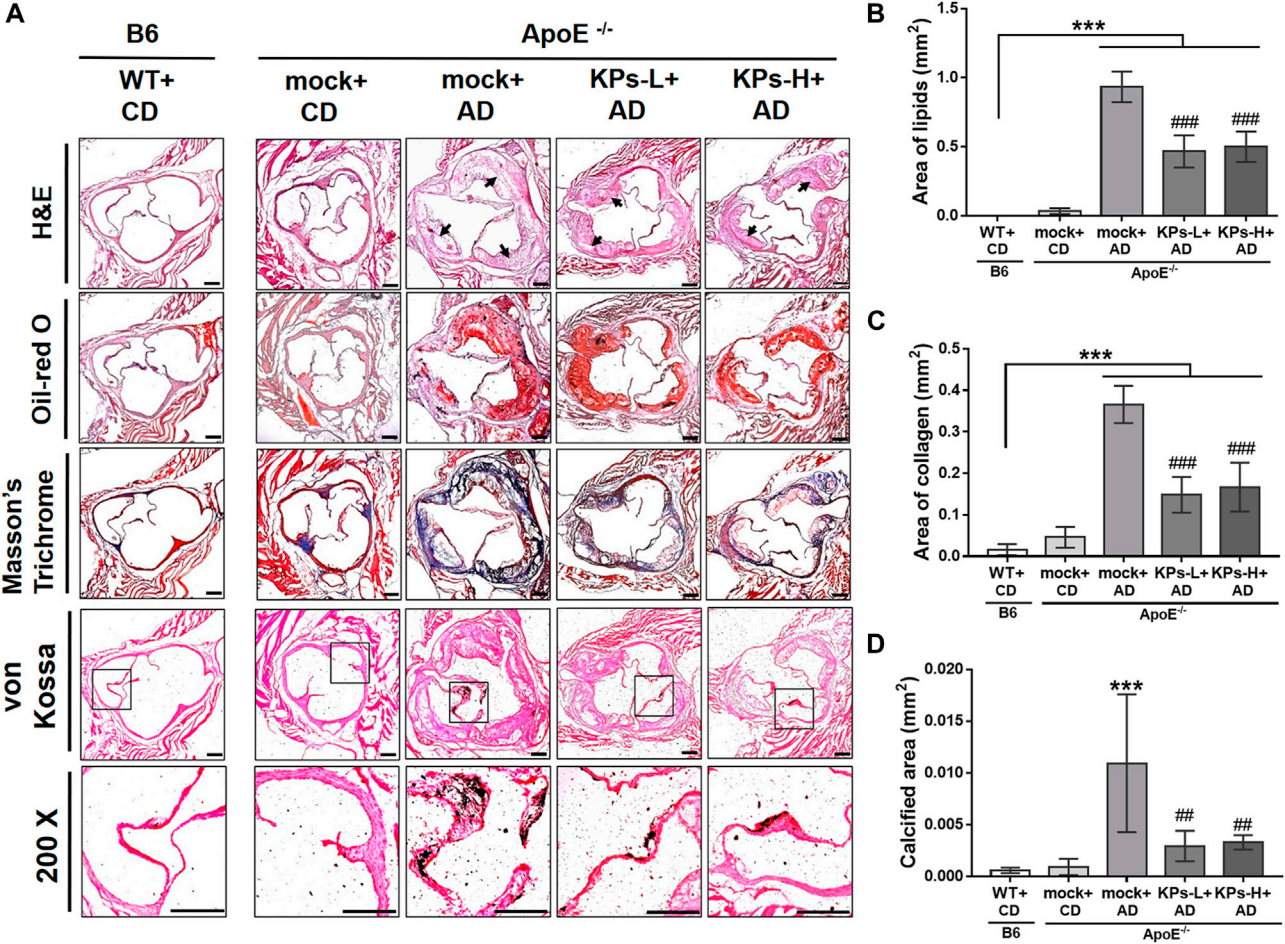
KPs attenuate the development of atherosclerotic vascular calcification in aortic roots. **(A)** Representative images of H&E, Masson’s trichrome, and von Kossa staining at the aortic roots. Arrows indicate aortic valves. **(B–D)** Area quantification of lipids, collagen, and calcification within the aortic roots. Scale bar: 200 μm. Statistical signs (*n* = 6): ***p < 0.001 vs. WT + CD/B6, and ^##^ *p* < 0.01, ^###^ *p* < 0.001 vs. mock + AD/*ApoE* ^*−/−*^.

We characterized the AD-induced pathological changes in aortic root tissue sections using several staining methods. As shown in Figure 2A, H&E images revealed apparent atherosclerotic plaques in the intima of the aortic root of the AD-fed *ApoE* ^*−/−*^ mice. We found that the oral administration of KPs hindered the deposition of atherosclerotic plaques in the aortic root. The observed atherosclerotic plaques were positively stained with Oil-red O dye, which indicated that abundant lipids were deposited in these plaques. The mock group of the AD-fed *ApoE* ^*−/−*^ mice exhibited the most significant lipid deposition compared to the other groups, but treatment with KPs ameliorated this pathological change to a significant extent (*p* < 0.001, Figure 2B). We further characterized the contents of collagen fibers in the aortic root using Masson’s trichrome staining. The results indicated that the highest content of collagen fibers was found in the mock group of AD-induced *ApoE* ^*−/−*^ mice, followed by KP-treated *ApoE* ^*−/−*^ mice, and the lowest content was found in normal B6 and CD-fed *ApoE* ^*−/−*^ mice (Figure 2C). Abnormal calcium deposits in the aortic root were identified using von Kossa’s method. The images indicated that calcification occurred predominantly in the aortic valves of the AD-induced *ApoE* ^*−/−*^ mice. The quantitative data were consistent with the other staining methods and showed the highest calcified areas in the mock group of the AD-fed *ApoE* ^*−/−*^ mice followed by the KP-treated *ApoE* ^*−/−*^ mice, and only background levels in normal CD-fed B6 and *ApoE* ^*−/−*^ mice (Figure 2D). However, none of the three staining methods revealed significant differences between low-dose and high-dose KP treatment groups (Figures 2B–D).

### KPs inhibit the inflammatory responses in the aortic root of AD-fed *ApoE^−/−^* mice

**FIGURE 3:**
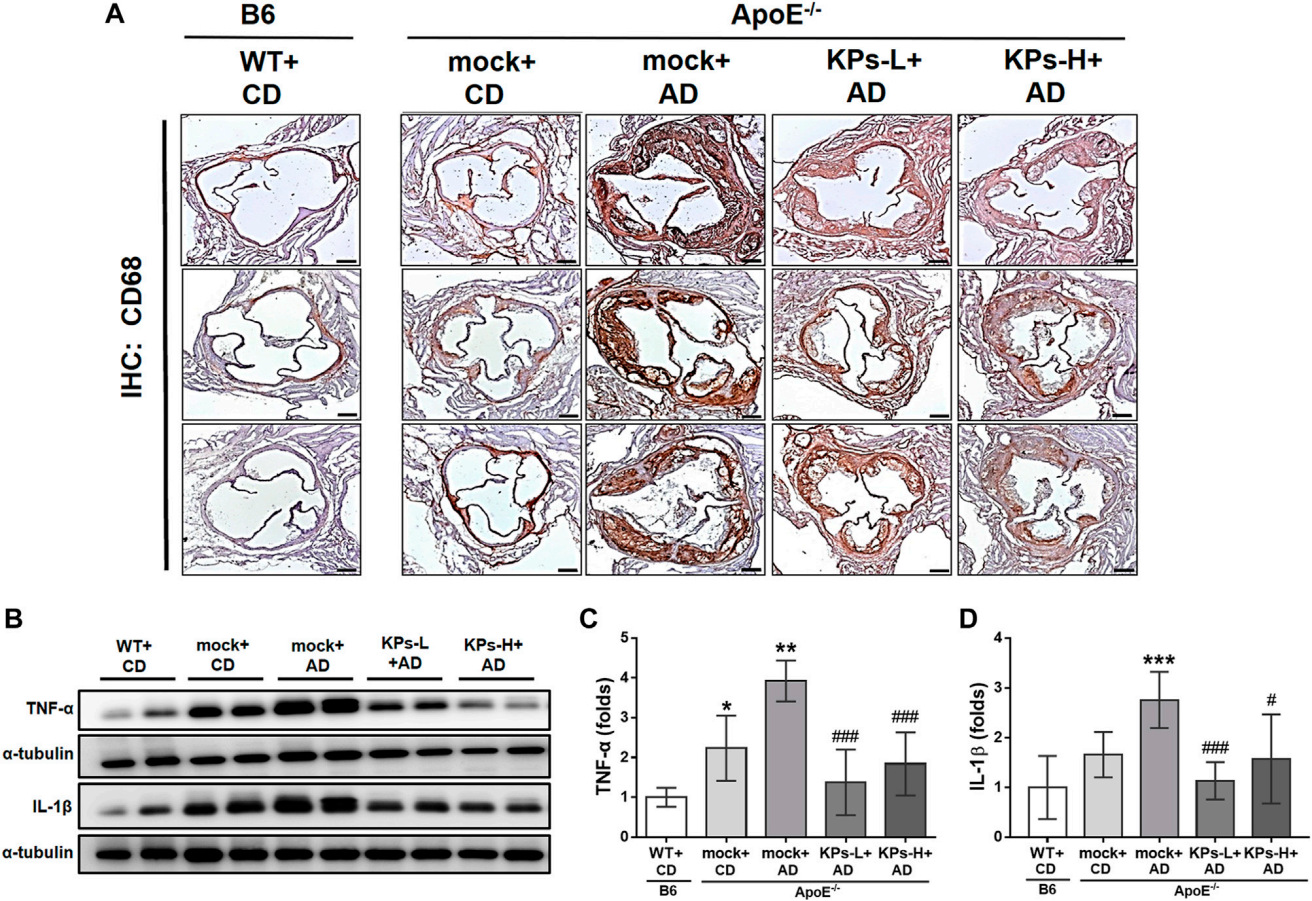
KPs attenuate local inflammatory responses in aortic roots. **(A)** Representative images of immunohistochemistry (IHC) staining against the surface marker of macrophages (CD68). Scale bar: 200 μm. **(B)** Western blot analysis of TNF-α and IL-1β expression in aortic root tissue lysates. The relative expression levels of TNF-α and IL-1β were quantified by normalizing to α-tubulin, as shown in **(C**, **D)**, respectively. Statistical signs (n = 6): *p < 0.05, **p < 0.01, ***p < 0.001 vs. WT + CD/B6, and ^#^ *p* < 0.05, ^###^ *p* < 0.001 vs. mock + AD/*ApoE* ^*−/−*^.

Because of the central role of inflammation in aortic calcification (Kawtharany et al., 2022), the expression of a classical macrophage marker, CD68, was investigated using IHC staining. As shown in Figure 3A, aortic tissues from the mock group of AD-fed *ApoE* ^*−/−*^ mice exhibited the highest CD68 expression among the groups. However, the expression of the CD68 marker was significantly inhibited in KP-treated *ApoE* ^*−/−*^ mice. Western blot analysis indicated that the mock group of AD-fed *ApoE* ^*−/−*^ mice also had significantly higher TNF-α and IL-1β expression than the other groups, and treatment with KPs significantly reduced TNF-α and IL-1β (Figures 3B–D). These results suggested that KPs inhibited inflammation in the aortic root of AD-fed *ApoE* ^*−/−*^ mice.

### KPs ameliorate bone loss in AD-fed *ApoE^−/−^* mice

**FIGURE 4:**
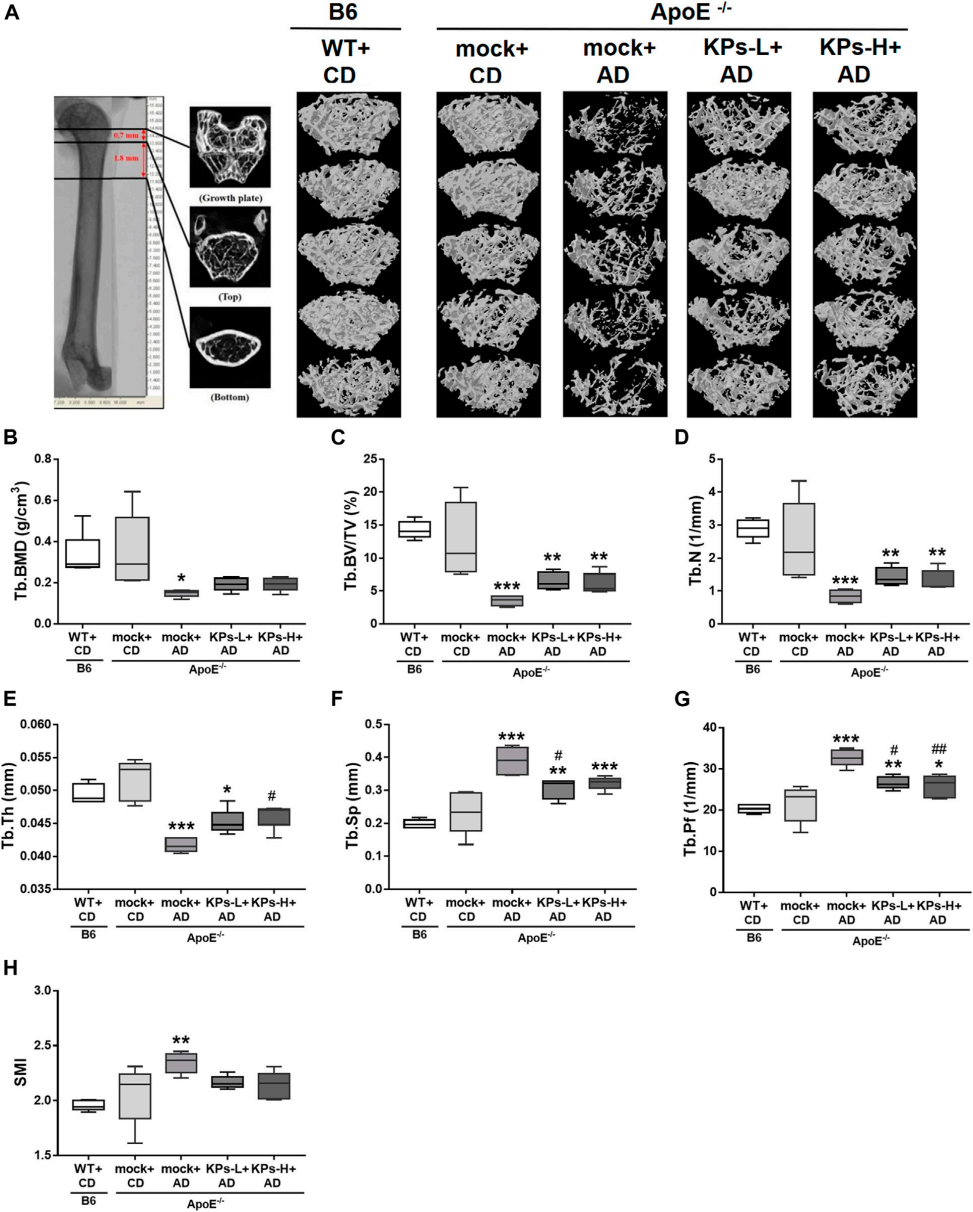
KPs prevent trabecular (Tb) bone loss in AD-fed *ApoE* ^*−/−*^ mice. The effects of KPs on femoral trabecular bones were evaluated using micro-CT. The reconstituted images of Tb structures are presented in a cross-section view **(A)**. The structural parameters are shown in **(B)** Tb. BMD, **(C)** Tb.BV/TV, **(D)** Tb.N, **(E)** Tb.Th, **(F)** Tb. Sp, **(G)** Tb. Pf, and **(H)** SMI. Statistical signs (*n* = 5): *p < 0.05, **p < 0.01, ***p < 0.001 vs. WT + CD/B6, and ^#^ *p* < 0.05, ^##^ *p* < 0.01 vs. mock + AD/*ApoE* ^*−/−*^.

Due to the negative correlation between vascular calcification and bone mineral density (BMD) (Persy and D'Haese, 2009), we further characterized the effects of AD and the oral administration of KPs on the changes in the related morphometric parameters of femoral bones. Micro-CT analysis of the femoral trabecular bones (Figure 4, Supplementary Figure S2) showed remarkable trabecular bone loss in the mock-treated AD-fed *ApoE* ^*−/−*^ mice (Figure 4A), with a decrease in the structural parameters Tb. BMD (*p* < 0.05), Tb.BV/TV (*p* < 0.001), Tb.N (*p* < 0.001), and Tb.Th (*p* < 0.001) and an increase in Tb. Sp (*p* < 0.001), Tb. Pf (*p* < 0.001), and SMI (*p* < 0.01) compared to the CD-fed B6 and *ApoE* ^*−/−*^ groups (Figures 4B–H). However, the KP-treated *ApoE* ^*−/−*^ groups exhibited a more compact trabecular structure (Figure 4A) and improved morphometric parameters of the trabecular bones (Figures 4B–H).

**FIGURE 5:**
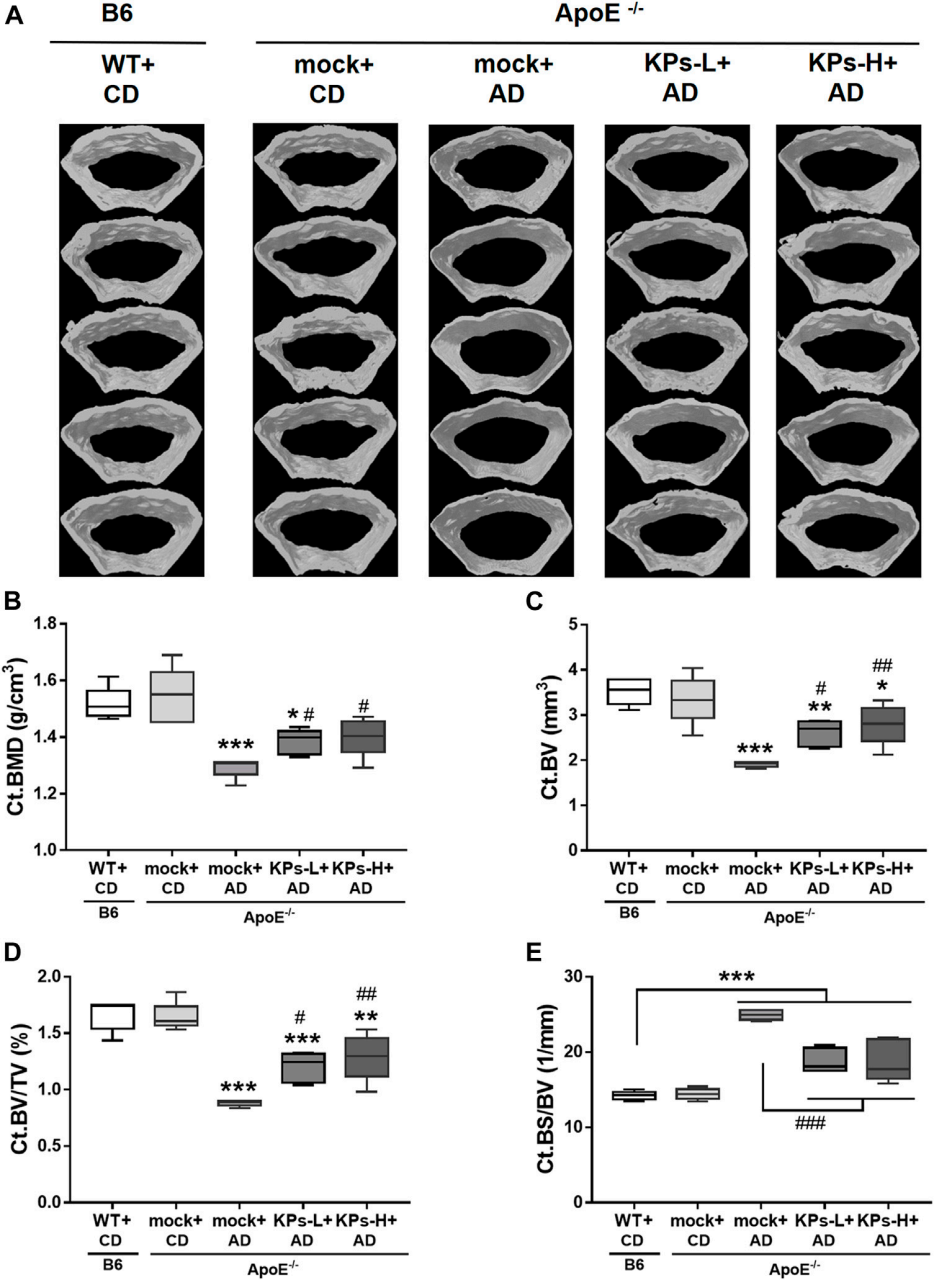
KPs increase cortical bone (Ct) volume in AD-fed *ApoE* ^*−/−*^ mice. The effects of KPs on femoral cortical bones were evaluated using micro-CT. **(A)** Reconstituted images of Ct structures from each mouse. The structural parameters of **(B)** Ct. BMD, **(C)** Ct. BV, **(D)** Ct. BV/TV, and **(E)** Ct. BV/SV are shown as indicated. Statistical signs (*n* = 5): *p < 0.05, **(*p* < 0.01, ***p < 0.001 vs. WT + CD/B6, and ^#^ *p* < 0.05, ^##^ *p* < 0.01, ^###^ *p* < 0.001 vs. mock + AD/*ApoE* ^*−/−*^.

AD also significantly affected the cortical bones of *ApoE* ^*−/−*^ mice (Figure 5A), as indicated by the decreases in the structural parameters Ct. BMD (*p* < 0.001), Ct. BV (*p* < 0.001), and Ct. BV/TV (*p* < 0.001) and an increase in Ct. BS/BV (*p* < 0.001) (Figures 5B–E). Oral treatment with KPs also caused less cortical bone loss in the AD-fed *ApoE* ^*−/−*^ mice (Figure 5A), which was reflected in the improved morphometric parameters of cortical bones (Figures 5B–E).

**FIGURE 6:**
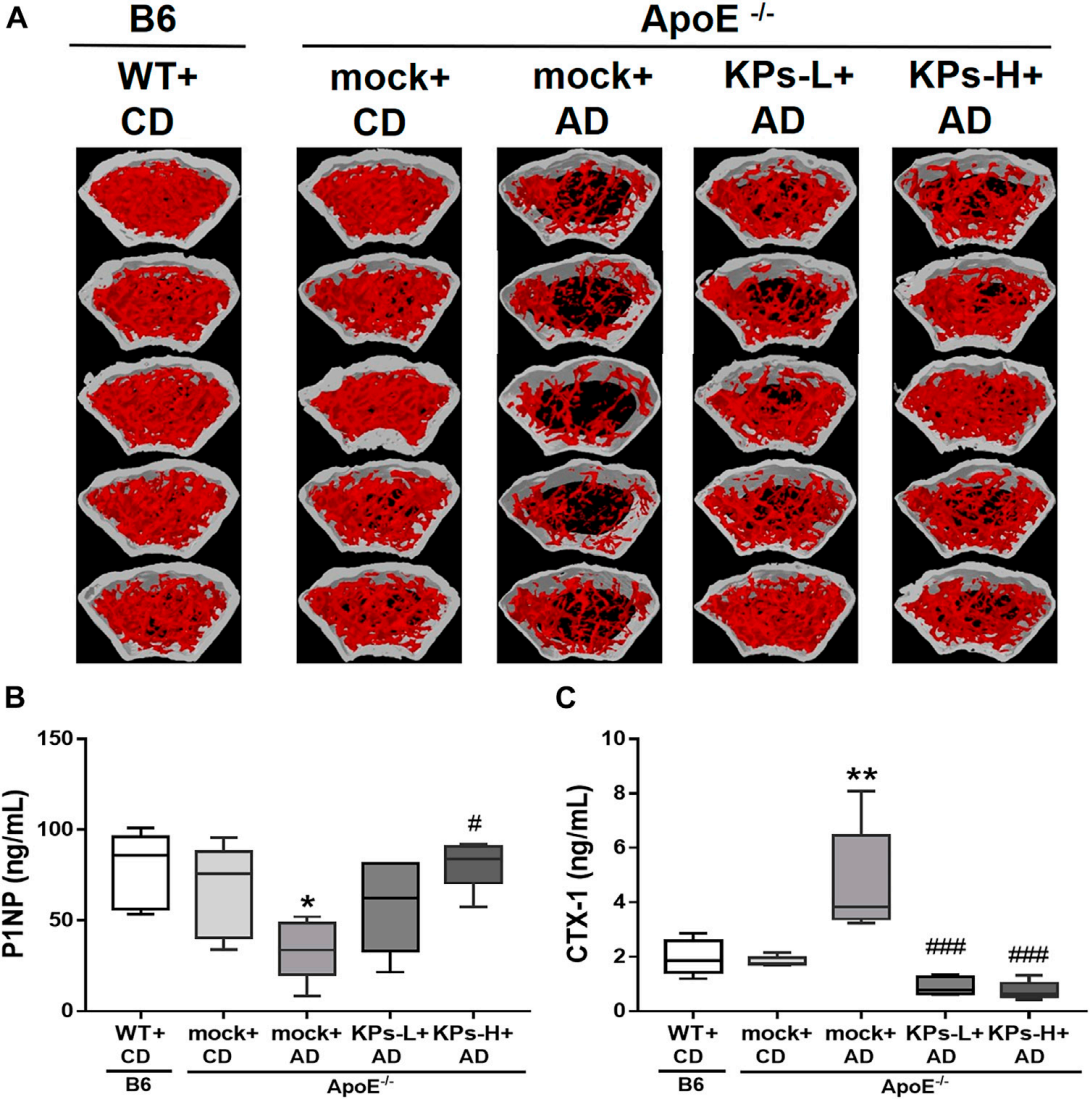
Effects of KPs on femurs **(A)** and on the serum bone formation marker P1NP **(B)** and resorption marker CTX-1 **(C)**. Statistical signs (*n* = 5): *p < 0.05, **p < 0.01 vs. WT + CD/B6, and ^#^ *p* < 0.05, ^###^ *p* < 0.001 vs. mock + AD/*ApoE* ^*−/−*^.

The combined results showed that KPs prevented AD-fed *ApoE* ^*−/−*^ mice from significant trabecular and cortical bone loss (Figure 6A). We further examined the changes in serum bone remodeling markers. The mock-treated AD-fed *ApoE* ^*−/−*^ mice showed lower expression of the bone formation marker P1NP (*p* < 0.05, Figure 6B) and higher expression of the bone resorption marker CTX-1 (*p* < 0.01, Figure 6C) than CD-fed B6 and *ApoE* ^*−/−*^ mice. KP treatment increased P1NP and reduced CTX-1 in KP-treated *ApoE* ^*−/−*^ groups, which exhibited comparable levels with CD-fed B6 and *ApoE* ^*−/−*^ mice (Figures 6B, C).

## Discussion

The present study found that an atherogenic diet with high cholesterol content caused dyslipidemia, which was followed by the development of atherosclerosis, VC, and osteoporosis in *ApoE* ^*−/−*^ mice as early as 20 weeks of age. The enhanced serum levels of ox-LDL, MDA, and proinflammatory cytokines, such as TNF-α, found in our work contributed to the increased oxidative stress and inflammation in the blood vessels of *ApoE* ^*−/−*^ mice with these implicated diseases. ApoE protein mediates the binding of lipoprotein particles in the circulation to specific lipoprotein receptors, which allows for the distribution and redistribution of lipids among various tissues and cells of the body. Early research showed that the lack of ApoE resulted in hyperlipidemia and atherosclerosis-like cardiovascular diseases in people (Schaefer et al., 1986) and animals (Plump et al., 1992; Zhang et al., 1992). Therefore, the *ApoE* ^*−/−*^ mouse model is frequently used for lipid metabolism and cardiovascular disease research (Emini Veseli et al., 2017). This model was recently used in research on the bone-vascular axis, and the results demonstrated a tight association between osteoporosis and cardiovascular diseases (Hjortnaes et al., 2010; Papachristou et al., 2018). However, the first study on the effects of ApoE on bone showed that ApoE deficiency increased bone formation in mice (Schilling et al., 2005). This finding may be explained by the fact that no high-fat or high-cholesterol diet was used in their experiments. We found no significant differences in bone phenotypes between wild-type and *ApoE* ^*−/−*^ mice fed a standard chow diet. Most studies demonstrated that high-fat or high-cholesterol diets induced osteoporosis in *ApoE* ^*−/−*^ mice (Sage et al., 2011; Liu et al., 2016; Yang et al., 2019; Luo et al., 2021). Hyperlipidemia is a shared risk factor for VC and osteoporosis. High-cholesterol diets increased serum total cholesterol levels but decreased serum triglycerides in *ApoE* ^*−/−*^ mice (Figures 1A,B). The reduction in serum triglycerides was likely due to the loss of ApoE function in hepatic VLDL secretion (Tsukamoto et al., 2000), which results in the accumulation of excess amounts of triglycerides in livers and subsequent liver damage, as evidenced by increased serum hepatic enzyme activities (Figures 1F–H). We discovered that high-cholesterol diets increased serum creatine kinase levels in *ApoE* ^*−/−*^ mice (Figure 1I), which suggests that hyperlipidemia also causes muscle damage. Consistent with other studies, our results suggested that systemic and local oxidative stress and inflammation stimulate VC and bone loss. The oxidative stress in our work comes from the oxidized lipids (e.g., ox-LDL) and lipoxidation end-products (e.g., MDA) (Figures 1C, D), which accumulate in the vasculature to trigger atherosclerosis and deposit in bone tissues to promote osteoporosis. It is clear from the current model that lipid-rich atherosclerotic plaques deposit on the intimal wall of the aortic root (Figure 2). These plaques have a high osteogenic factor content of collagen and show characteristics of atherosclerotic calcification (Figure 2). Predominant CD68 macrophage marker levels and increased IL-1β and TNF-α were detected (Figure 3), which suggested local inflammation in the aortic roots. In addition to vascular damage, hyperlipidemia-induced bone damage was also profound in the present study, which was demonstrated by the changes in serum bone formation (P1NP) and bone resorption (CTX-1) markers (Figure 6) and considerable bone loss in femoral trabecular and cortical bones (Figures 4, 5).

Although the underlying mechanisms that manipulate the bone-vascular axis remain obscure, the implications of oxidative stress and inflammation provide us with new insights. For example, Sage et al. demonstrated that oxidized lipids reduced osteogenesis and influenced the bone anabolic effects of parathyroid hormone (PTH) by encouraging bone resorption (Sage et al., 2011), which suggests the resistance to PTH therapy in osteoporotic patients with hyperlipidemia. *In vitro*, Song et al. demonstrated that ox-LDL induced the calcification of human vascular smooth muscle cells (VSMCs) *via* Toll-like receptor 4 (TLR4), which highlighted the critical role of TLR4 and inflammation-related NK-κB signaling in VC (Song et al., 2017). Hong et al. showed that long-term exposure to ox-LDL reduced the expression of Sirt1 (aging-related marker) and Runx2 (mediator of bone formation) in bone marrow-derived mesenchymal stem cells, which explained why aged *ApoE* ^*−/−*^ mice had less bone mass than age-matched C57Bl/6J mice (Hong et al., 2015). Oxidative stress initiates the inflammatory process, which results in the secretion of proinflammatory cytokines in many chronic diseases. Proinflammatory cytokines, including TNF-α, IL-1β and IL-6, activate *in vitro* and *in vivo* osteoclast activity in bone *via* the NK-κB pathway (Lin et al., 2017). However, these inflammation-related cytokines and NK-κB signaling mediate calcification in VSMCs and experimental mouse models by downregulating Klotho (antiaging factor) in the kidney and Fetuin A (mineralization inhibitor) in the liver (Henaut et al., 2016). Natural products targeting the adverse effects of oxidative stress and inflammation in the vasculature and bone tissues may provide new therapeutic strategies for the resolution of atherosclerotic VC and bone deterioration.

The present study found that oral treatment with KPs exerted notable lipid-lowering activity in AD-fed *ApoE* ^*−/−*^ mice, specifically the modulation of systemic ox-LDL levels (Figure 1C). Although systemic total cholesterol levels in KP-treated *ApoE* ^*−/−*^ mice continued to be higher than normal, the lowering effect remained significant compared with AD-fed *ApoE* ^*−/−*^ mice without KP treatment (Figure 1A). KP treatment substantially decreased the lipid contents of the atherosclerotic plaques around the aortic roots (Figure 2). The lipid-lowering effects exclude the factors of diet and total energy intake because there was no discernible difference in the final weight gain and similar diet and total energy intake were recorded in AD-fed *ApoE* ^*−/−*^ mice (Supplementary Figure S1). We showed previously that KPs effectively prevented hyperlipidemia and obesity in high-fat diet-induced obese rats by reducing fatty acid synthase and increasing p-acetyl-CoA carboxylase (p-ACC) expression to block lipogenesis and decreasing inflammatory responses (TNF-α, IL-1β, and TGF-β) to attenuate oxidative damage in the liver (Tung et al., 2018). Despite the use of two distinct animal models in our studies, the outcomes were consistent. The reduction in total cholesterol, ox-LDL, and the end-products of lipid peroxidation (MDA) in *ApoE* ^*−/−*^ mice treated with KPs was accompanied by a lower systemic TNF-α level (Figure 1E) and attenuated local inflammatory responses (CD68, TNF-α, IL-1β) in the aortic tissues (Figure 3). We found that KP treatment slowed the progression of aortic calcification in *ApoE* ^*−/−*^ mice, which was evidenced by decreased collagen expression and calcified areas around the aortic roots (Figure 2). Our previous work showed that KPs improved the circulating lipid profile by increasing “good cholesterol” HDL and decreasing “bad cholesterol” LDL and ox-LDL and protected against aortic atherosclerosis by decreasing oxidative stress (serum ROS, nitric oxide, ox-LDL) and local inflammatory responses (MOMA-2, MCP-1, TNF-α, IL-1β) in high-fat diet (HFD)-fed *ApoE* ^*−/−*^ mice (Tung et al., 2020). KPs also inhibited ICAM-1 expression, which is a critical endothelial marker for the recruitment of proinflammatory macrophages to atherosclerotic sites. Notably, the mean cholesterol level induced by AD in the current study was 4 times higher than our previous HFD-fed model, which promoted atherosclerosis to further deteriorate into VC and aggravate the severity of cardiovascular diseases.

Concomitantly, KP treatment improved BMD and microarchitecture in trabecular (Figure 4) and cortical bones (Figure 5) with an increase in the circulating bone formation marker P1NP and a reduction in the resorption marker CTX1 (Figure 6), which showed that bone remodeling favored bone formation in KP-treated *ApoE* ^*−/−*^ mice. In fact, KPs have repeatedly demonstrated bone-protective benefits in our earlier animal models of osteoporosis caused by estrogen depletion (Chen et al., 2015; Tu et al., 2020), hemophilia (Yen et al., 2022), and vitamin C insufficiency (Chang et al., 2022), and rats with glucocorticoid-induced osteoporosis (de Vasconcelos et al., 2022). We recently demonstrated that KPs promoted osteoblastic differentiation and bone mineralization while inhibiting RANKL-induced osteoclastic differentiation and bone resorption *in vitro* (Chang et al., 2022), and treatments with KPs eventually lowered the circulating RANKL/OPG ratio *in vivo* (Chang et al., 2022; Yen et al., 2022). Although serum RANKL and OPG levels were not measured at the end of this study, it was speculated that KP treatment could lower the serum RANKL/OPG ratio in the current *ApoE* ^*−/−*^ model. In fact, many anti-osteoporotic agents with excellent anti-inflammatory activity were also shown to decrease the serum RANKL/OPG ratio (Niwano et al., 2022; Marcucci et al., 2023). As a key regulator of bone remodeling, OPG prevents RANKL from binding to its receptor RANK, which restricts osteoclast differentiation, maturation, and survival while promoting bone formation (Zhao et al., 2016). RANKL and OPG are expressed in atherosclerotic lesions of humans and mice (Di Bartolo et al., 2013) and play important roles in VC (Collin-Osdoby, 2004). Endogenous OPG protected *ApoE* ^*−/−*^ mice from atherosclerosis and VC (Callegari et al., 2013), and the transgenic expression of RANKL in VSMCs promoted VC in mice (Morony et al., 2012). Consequently, we thought that KPs could regulate the RANKL/OPG axis to prevent VC and osteoporosis concomitantly. However, this still requires further verification.

Therapies targeting inflammation may impact VC and osteoporosis, and several therapeutic monoclonal antibodies targeting proinflammatory cytokines have been developed. Canakinumab (targeting IL-1β) reduced recurrent cardiovascular events without changing lipid profiles in the Canakinumab Anti-inflammatory Thrombosis Outcome Study (CANTOS) (Ridker et al., 2017). Subsequent analysis from the same study suggested that canakinumab reduced the rates of total hip and knee replacement and the associated osteoarthritic symptoms during the follow-up period of 3.7 years (Schieker et al., 2020). However, the limitation of canakinumab is a significantly increased risk of fatal infection and sepsis (Ridker et al., 2017). Another clinical trial revealed that denosumab (targeting RANKL) was effective in recovering aortic arch calcification in patients undergoing long-term hemodialysis. However, treatment with denosumab is expansive and carries the risks of severe refractory hypocalcemia (Suzuki et al., 2021). Biologic agents may be worth investigating, but these agents are expensive and may have adverse effects.

Kefir and its associated peptides have raised interest in the scientific community due to their safety, ease of production, lower cost, and numerous beneficial effects on health, including cholesterol-lowering, antihypertensive, anticarcinogenic, and antimicrobial effects and antioxidant and anti-inflammatory activities (Pimenta et al., 2018). In prosperous countries, aging and increasing chronic inflammatory cardiovascular and bone-related diseases are inevitable. Fighting for these issues is undoubtedly the main challenge for preventive medicine.

In conclusion, KPs act against hyperlipidemia, oxidative stress, and inflammation to halt the progression of atherosclerotic VC and osteoporosis, which may be one of the holistic solutions for bone-vascular axis and aging issues. The present study advances our knowledge of the effectiveness of kefir and proposes the clinical use of KPs to avoid the onset of chronic cardiovascular illnesses and related bone deterioration. Recently, the Kusumbe lab exploited cutting-edge 3D tissue imaging techniques to dissect blood and lymphatic vessel changes in single-cell resolution to uncover the interactions between vascular and immune cells over time and across different tissues and organs, in which they proposed that the age-dependent vascular and perivascular changes are caused by endothelial cell inflammation and a variety of genes and signals that eventually lead to vascular loss and fibroblast accumulation (Chen et al., 2021). Later, they reported that genotoxic stress-induced IL-6 drives the expansion of lymphatic vessels (lymphangiogenesis) in bone and causes the proliferating lymphatic endothelial cells to secrete CXCL12 to trigger the expansion of Myh11^+^CXCR4^+^ pericytes, which contribute to bone and hematopoietic regeneration (Biswas et al., 2023). These studies shed light on the research of age-related vascular and bone diseases in the future and may help us to unveil the molecular mechanisms behind the efficacy of KPs and to identify the potential bioactive peptide candidates in preventing VC and osteoporosis simultaneously.

## Data Availability

The original contributions presented in the study are included in the article/Supplementary Material, further inquiries can be directed to the corresponding authors.

## Supplementary material

SUPPLEMENTARY FIGURE S1 Effects of KPs on changes in body weight **(A)**, diet **(C)** and energy intake **(D)**. The body weights and diet intakes were recorded every week. The energy intakes were calculated by the combination of total energy from diet (CD: 3.227 kcal/g; AD: 4.05 kcal/g) and KPs (4.833 kcal/g). Body weight gain was obtained by calculating the difference in body weights between weeks 0 and 13, as shown in (B). Statistical signs (*n* = 6): ****p* < 0.001 (WT+CD vs. mock+CD) and ^###^ *p* < 0.001 (CD vs. AD).

SUPPLEMENTARY FIGURE S2 Effects of KPs on serum **(A)** ALT, **(B)** AST, **(C)** ALT/AST, and **(D)** CK levels. The dashed lines indicate the normal ranges of target serum markers in mice. Statistical signs (*n* = 6): **p* < 0.05, ***p* < 0.01, ****p* < 0.001 vs. WT+CD/B6, and ^#^ *p* < 0.05, ^##^ *p* < 0.01, ^###^ *p* < 0.001 vs. mock+AD/*ApoE−/−*.

SUPPLEMENTARY FIGURE S3 A longitudinal-view of femoral trabecular (Tb) bones was evaluated using micro-CT (*n* = 5).

SUPPLEMENTARY FIGURE S4 Western blots raw data of Figure 3B.

## Abbreviations

KPs, Kefir peptides; VC, vascular calcification; ApoE, apolipoprotein E; AD, atherogenic diet; CD, chow diet; LDL, low-density lipoprotein; ox-LDL, oxidized-LDL; HDL, high-density lipoprotein; MDA, malondialdehyde; ALT, alanine aminotransferase; AST, aspartate aminotransferase; CK, creatine kinase; TNF-α, tumor necrosis factor-alpha; IL, interleukin; NF-κB, nuclear factor kappa-light-chain-enhancer of activated B cells; RANKL, receptor activator of nuclear factor kappa-B ligand; OPG, osteoprotegerin; P1NP, procollagen type I intact N-terminal propeptide; CTX-1, carboxyl-terminal cross-linked telopeptide of type I collagen.
