# Supplementary figures and images for: Kefir peptides attenuate atherosclerotic vascular calcification and osteoporosis in atherogenic diet-fed ApoE −/− knockout mice
Source: Front Cell Dev Biol. 2023 Apr 6;11:1158812. doi: 10.3389/fcell.2023.1158812 (PMC10117689; doi:10.3389/fcell.2023.1158812)

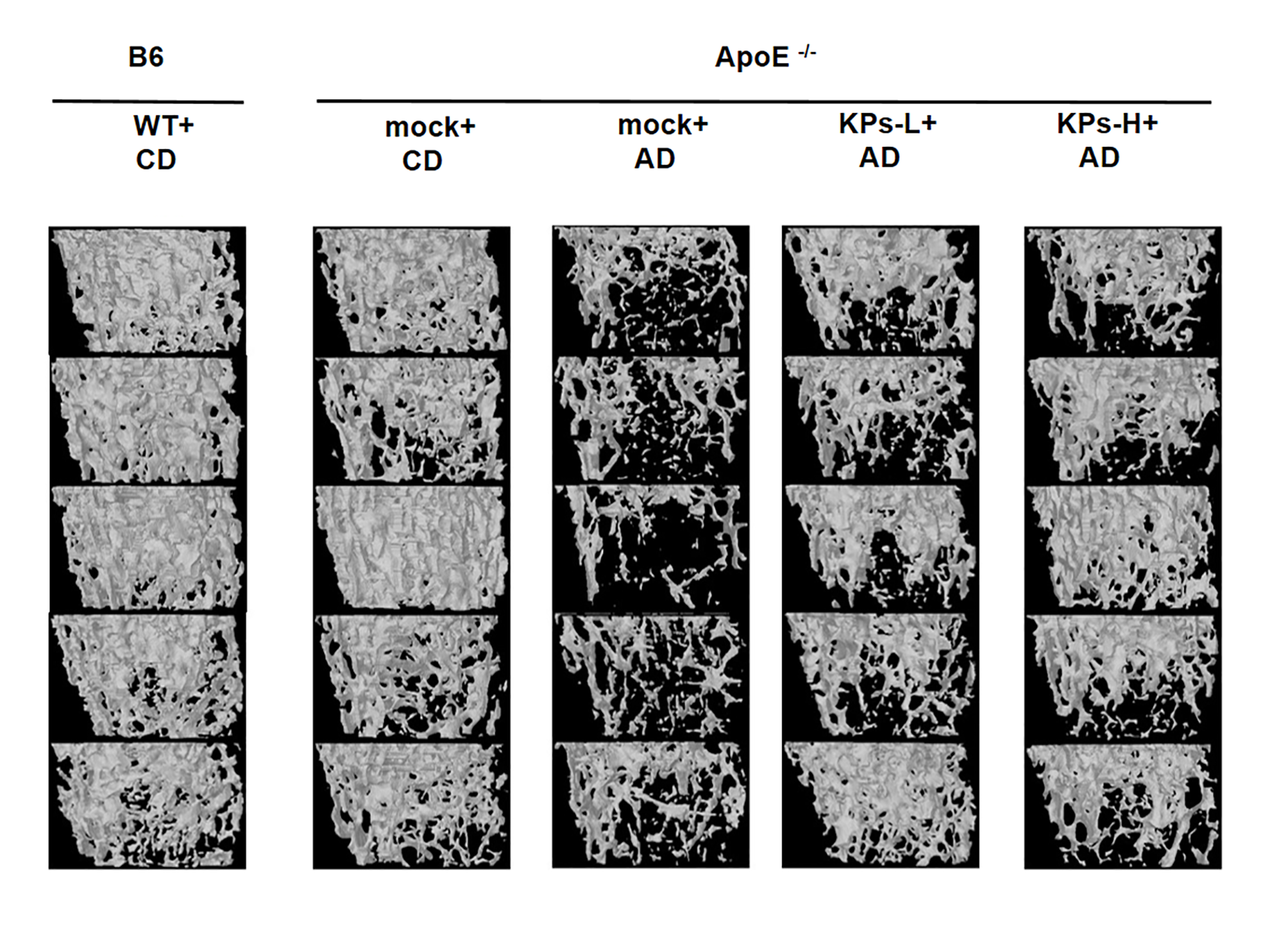

Supplement: Supplementary file 1 [file Image3.tif]

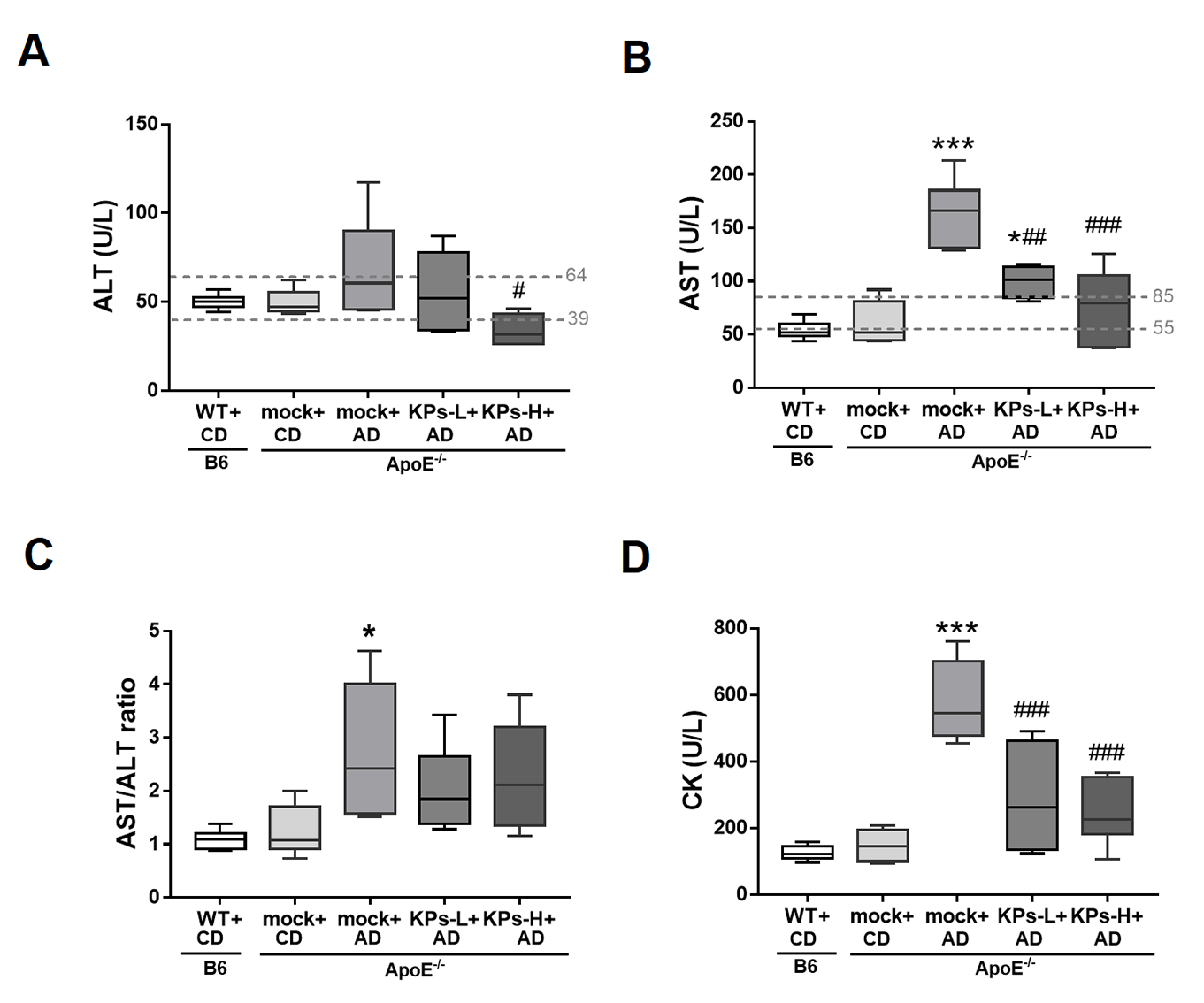

Supplement: Supplementary file 2 [file Image2.tif]

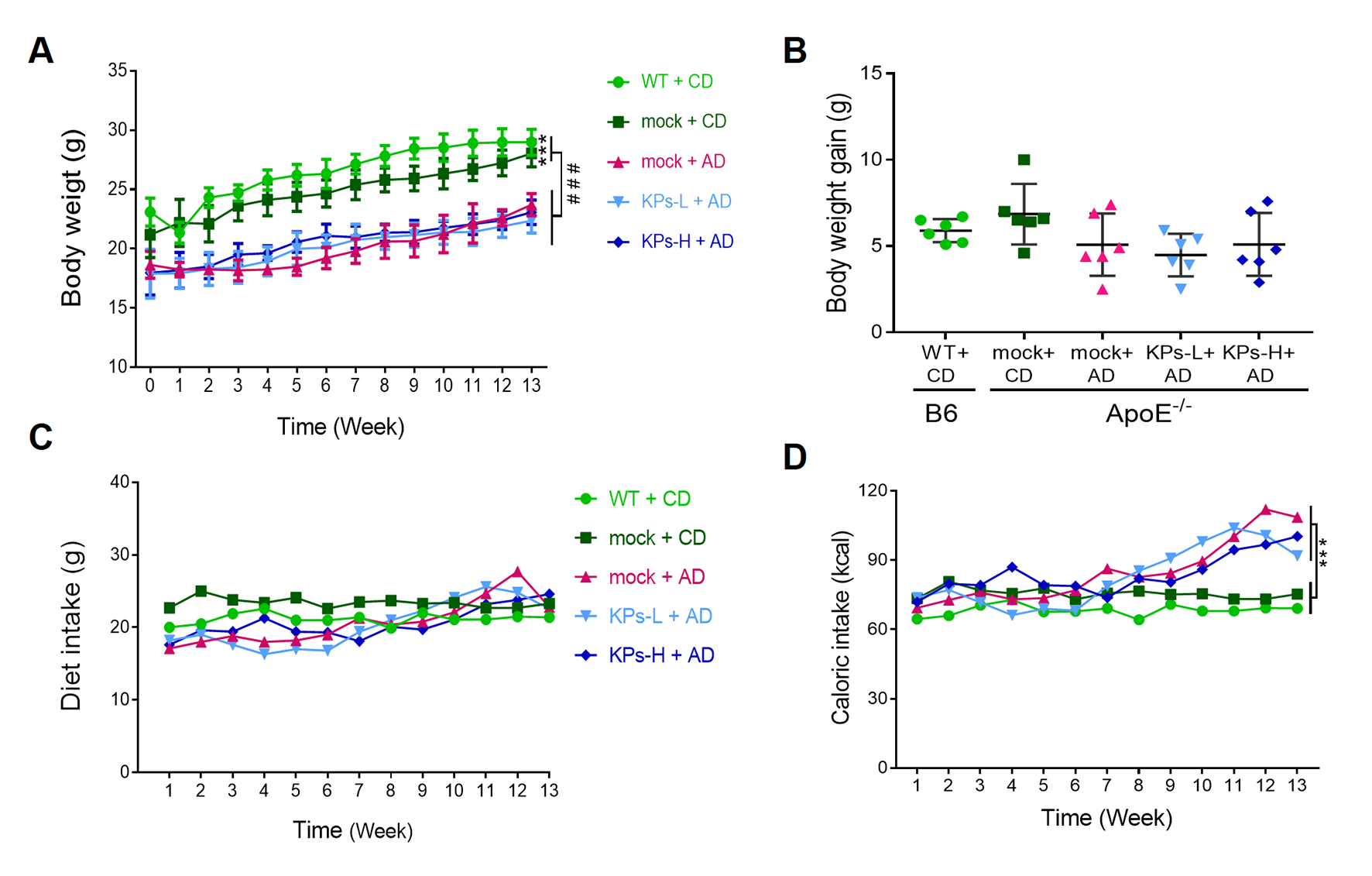

Supplement: Supplementary file 3 [file Image1.tif]
